# Assessing postural stability via the correlation patterns of vertical ground reaction force components

**DOI:** 10.1186/s12938-016-0212-z

**Published:** 2016-08-02

**Authors:** Chih-Yuan Hong, Lan-Yuen Guo, Rong Song, Mark L. Nagurka, Jia-Li Sung, Chen-Wen Yen

**Affiliations:** 1 Department of Mechanical and Electromechanical Engineering, National Sun Yat-Sen University, Kaohsiung, Taiwan; 2 Department of Sports Medicine, Kaohsiung Medical University, Kaohsiung, Taiwan; 3 School of Engineering, Sun Yat-Sen University, Guangzhou, China; 4 Department of Mechanical Engineering, Marquette University, Milwaukee, WI USA; 5 Department of Physical Therapy, Kaohsiung Medical University, Kaohsiung, Taiwan

**Keywords:** Ground reaction force, Postural balance, Quiet standing, Force plate

## Abstract

**Background:**

Many methods have been proposed to assess the stability of human postural balance by using a force plate. While most of these approaches characterize postural stability by extracting features from the trajectory of the center of pressure (COP), this work develops stability measures derived from components of the ground reaction force (GRF).

**Methods:**

In comparison with previous GRF-based approaches that extract stability features from the GRF resultant force, this study proposes three feature sets derived from the correlation patterns among the vertical GRF (VGRF) components. The first and second feature sets quantitatively assess the strength and changing speed of the correlation patterns, respectively. The third feature set is used to quantify the stabilizing effect of the GRF coordination patterns on the COP.

**Results:**

In addition to experimentally demonstrating the reliability of the proposed features, the efficacy of the proposed features has also been tested by using them to classify two age groups (18–24 and 65–73 years) in quiet standing. The experimental results show that the proposed features are considerably more sensitive to aging than one of the most effective conventional COP features and two recently proposed COM features.

**Conclusions:**

By extracting information from the correlation patterns of the VGRF components, this study proposes three sets of features to assess human postural stability during quiet standing. As demonstrated by the experimental results, the proposed features are not only robust to inter-trial variability but also more accurate than the tested COP and COM features in classifying the older and younger age groups. An additional advantage of the proposed approach is that it reduces the force sensing requirement from 3D to 1D, substantially reducing the cost of the force plate measurement system.

## Background

Postural control, the foundation of our ability to stand and walk, is a complex motor skill derived from the interaction of multiple sensorimotor processes. Deterioration of postural control can lead to balance impairment and has been found to be associated with the risk of falling [1–5]. Since fall is a major cause of morbidity and mortality for older people [6, 7], there is a crucial need for a simple-to-implement method to assess postural stability in order to predict fall risk.

Conventionally, postural stability has been assessed by static and/or dynamic posturography [8–11]. Dynamic posturography evaluates postural stability by disrupting a stable stance and measuring the postural response to the external perturbation. In contrast, static posturography measures the postural steadiness of a subject standing quietly (typically on a force plate) without any external disturbance. Numerous experimental studies suggest that subjects who exhibit larger sway during quiet standing have poorer postural stability [5, 12–16].

Mechanically, balance is achieved when the external forces and torques applied to a body are in static equilibrium. During quiet standing, there are two external forces: the weight and the ground reaction force (GRF). The weight acts at the center of mass (COM), which is the point where all of the mass of the human body can be considered to be concentrated. The GRF acts the center of pressure (COP), which is the point of application of the resultant GRF below the body. With these two external forces, any difference between the vertical projection of the COM and the COP results in a moment that destabilizes postural balance [17, 18]. To avoid postural instability, the COP regulates the COM by adjusting the location of the vertical projection of COM to maintain it within a region between the two feet (the polygon of support).

By modelling the standing body be as an inverted pendulum pivoting about ankle, the postural balance system has often been viewed as a feedback control system with the COM and COP as the output and control variables, respectively [17]. The time responses of the COP and COM have been used to provide insights into the control mechanism of human balance. Determining the COM is much more difficult than measuring the COP, which can be found easily by a force plate. As such, many studies have employed COP features to characterize the postural stability during quiet standing [19–24].

Since force plates measure the GRF, a number of studies have also tried to use GRF-based features to assess postural stability. For example, it was found that there was a significant correlation between the standard deviation of the vertical component of the GRF (VGRF) and the Berg Balance Scale (BBS) score [25], which is a 14-item validated scale that evaluates balance abilities during sitting, standing and positional changes. A previous study found that young adults tend to have larger GRF variability than older adults during the first 5 s of single leg stance [15]. For post-stroke patients, it was shown that the standard deviation of the anterior–posterior GRF component is the most prominent feature that correlates to the BBS score [26]. Statistically significant differences in the VGRF were also found between Parkinson patients and healthy controls [27]. After reviewing thirteen articles that studied the relationship between the history of lower-extremity stress fractures and GRF, the loading rate of the VGRF was concluded to be different for patient and control groups [28]. In summary, the results of previous studies demonstrate the importance of the GRF and its utility in assessing postural stability.

In interacting with the environment and objects, humans typically make multiple adjustments of forces that require agile and subtle coordination. For example, in multi-fingered grasping of an object the forces applied by the digits are highly coordinated to achieve precise positioning and to guarantee that the needed net force is applied. A challenge for performing a coordination task is to determine the distribution of the applied forces. This has been identified as a motor redundancy problem (also known as Bernstein’s problem) since the resultant grasping force can be achieved with an infinite number of combinations of individual finger forces when the number of constraints of the grasping task is smaller than the number of the employed fingers [29].

In attempting to discover the strategies behind multi-finger force production, recent experimental results suggest that forces exerted by all digit pairs tend to be synchronized under a variety of task constraints [30, 31]. Studies have also found that the strength and consistency of finger force covariation may vary with task [32, 33], age [34–36], magnitude of the total force [37] and disease, such as stroke [38], carpal tunnel syndrome [39], olivo-ponto-cerebellar atrophy [40] and hemiplegic cerebral palsy [41].

A force plate, one of the most popular tools for quantitative assessment of postural stability, is a rectangular plate with force transducers that measure the applied force [42]. In similarity to finger forces that need to be coordinated with one another to sum to the total needed force, the measurements from the force transducers, which are typically located at the four corner of the force plate, also need to sum to the total GRF. This similarity can be demonstrated by comparing the task of holding a cup and the event of standing on a force plate. Just as the weight of the cup must be shared by the four fingers that are holding it, the weight of the human body must be balanced by the four GRF components measured by the force plate. A fundamental difference between these two weight sharing tasks is that finger forces are directly generated and controlled by human hands whereas the GRF components are indirectly produced and controlled by human body motion. Despite such a difference, since both tasks require the coordination of multiple human-generated forces, it is valuable to investigate whether the results of extensive studies on the multi-digit force coordination problems can help us understand the coordination mechanism of the GRF components during postural balance. Considering the sensitivity of the inter-finger force coordination patterns to aging and other operating and physiological conditions, it seems reasonable to assume that there are associations between the GRF component coordination patterns and postural stability.

Compared to previous GRF based postural balance studies that extract stability features from the GRF resultant, this work looks into the components of the GRF. In particular, this work proposes features to characterize the strength, the changing speed and the stabilizing effect of the coordination patterns of the GRF components. To the best of our knowledge, this is the first attempt to use the GRF coordination properties to assess postural balance. The hypothesis is that the proposed features can detect the aging-related changes in postural balance. The promise of this work is that the proposed features can help uncover ways in which the postural control system is compromised with the aging process and thereby provide valuable information in identifying persons at risk of falling.

## Methods

### Subjects

The tested subjects consisted of two adult age groups: an older age group (68.7 ± 2.96 years, range 65–73 years, BMI 23.9 ± 4.13 kg/m^2^) and a younger age group (20.1 ± 1.29 years, range 18–24 years, BMI 22.5 ± 3.21 kg/m^2^). Each group consisted of ten male and ten female healthy adults, none of whom had pathological conditions that would compromise their postural performance. All experimental procedures were approved by the Institutional Review Board of the Kaohsiung Medical University Chung-Ho Memorial Hospital, Kaohsiung, Taiwan.

### Measurements

**Fig. 1:**
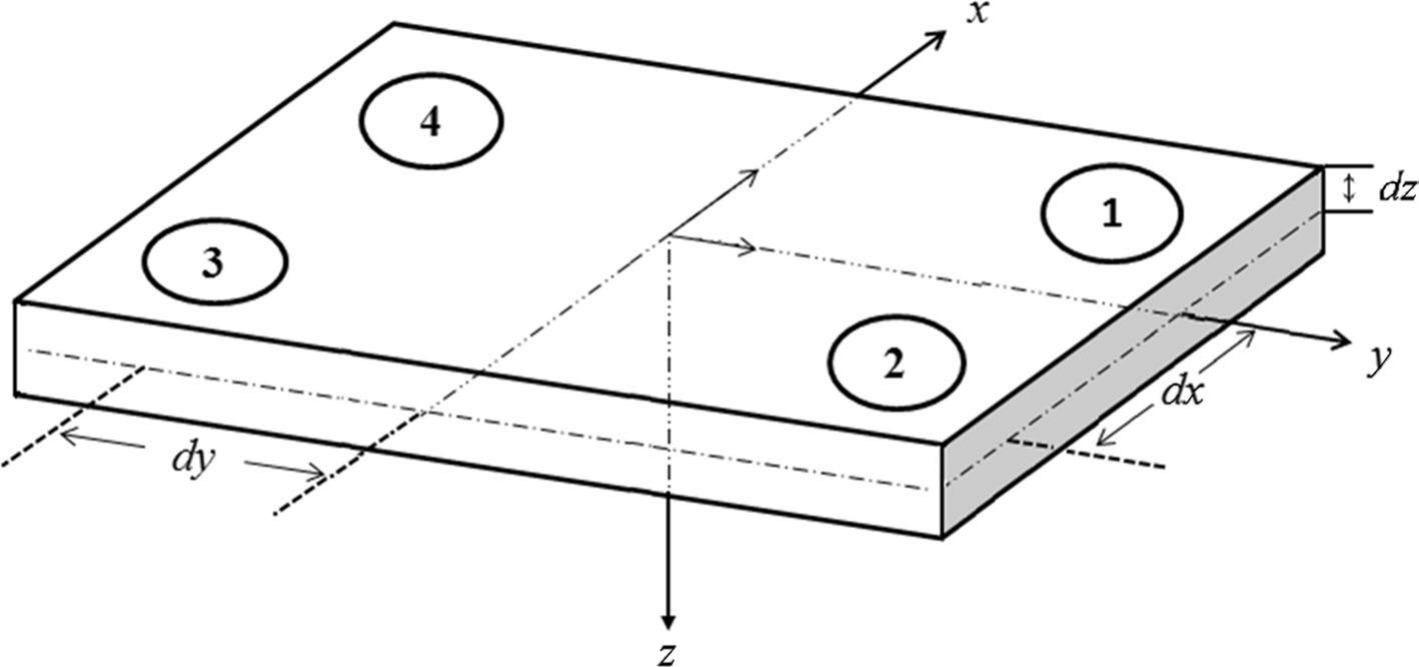
A force plate, its coordinate system, and locations of four force transducers

For a conventional force plate, shown in Fig. 1, load cells or piezoelectric force transducers are positioned at the four corners. In Fig. 1, the *x*, *y* and *z* axes correspond to the anterior–posterior (AP), medial-lateral (ML) and vertical directions, respectively. The origin of the coordinate system is located at the center of the force plate. With ***F***_*i*_ signifying the force vector at the *i*th corner of the force plate, the GRF vector (denoted by ***f***) can be expressed as

1 $${\mathbf{f}} = f_{x} {\user2{i}} + f_{y} {\user2{j}} + f_{z} {\user2{k}} = {\user2{F}}_{1} + {\user2{F}}_{2} + {\user2{F}}_{3} + {\user2{F}}_{4}$$

where ***i***, ***j*** and ***k*** are unit vectors along the *x*, *y* and *z* axes and *f*_*x*_, *f*_*y*_ and *f*_*z*_ represent the GRF components in the AP, ML and vertical directions, respectively. Denoting the vertical component of ***F***_*i*_, as *F*_*iz*_,

2 $$f_{z} = F_{1z} + F_{2z} + F_{3z} + F_{4z}$$

Hereafter, we refer to *f*_*z*_ as VGRF and *F*_1*z*_, *F*_2*z*_, *F*_3*z*_ and *F*_4*z*_ as VGRF components at the respective corners. Next, by defining *F*_*ijα*_ as the sum of *F*_*iα*_ and *F*_*jα*_ (*i*, *j* = 1, 2, 3 or 4 and *α* = *x, y* or *z*), the following equations of the COP coordinates can be derived by using Newtonian mechanics:

3 $$COP_{x} = \frac{{d_{x} (F_{14z} - F_{23z} ) + d_{z} f_{x} }}{{f_{z} }}$$

4 $$COP_{y} = \frac{{d_{y} (F_{12z} - F_{34z} ) + d_{z} f_{y} }}{{f_{z} }}$$

As shown in Fig. 1, *d*_*x*_ and *d*_*y*_ represent the distances along the *x*-axis and *y*-axis, respectively, from the coordinate axes to the force sensors, and *d*_*z*_ represents the distance along the *z*-axis from the coordinate system to the support surface of the force plate.

In this work, the measurement system consists of a force plate (Kistler 9286AA) connected to a PC-based signal processing system. This force plate system can provide the following signals: two COP coordinates (*COP*_*x*_ and *COP*_*y*_) and eight GRF components (*F*_1*z*_, *F*_2*z*_, *F*_3*z*_, *F*_4*z*_, *F*_12*x*_, *F*_34*x*_, *F*_14*y*_ and *F*_23*y*_), all of which were recorded at a sampling rate of 512 Hz and filtered by a sixth-order Butterworth filter with a cutoff frequency of 5 Hz. The data acquisition and display software was a custom program written in LabVIEW (National Instruments).

Each subject was tested on 2 days with two experimental sessions performed each day. Each session included three 80 s eyes-open-closed trials, during which the participants were barefoot and instructed to look straight ahead at a visual reference and stand quietly (with arms at the side) in a comfortable stance near the center of the force plate for the first 40 s and then maintaining the same posture the subjects closed their eyes in the remaining 40 s of the trial. The data collected from 5 to 35 s of the trials were used for this study. Trials and sessions were separated by approximately one and 5 min of rest, respectively. In summary, 240 (20 subjects × 3 trials × 4 sessions) measures were collected for both age groups. This work focuses on the eyes-open data.

### Proposed postural stability features

By analyzing the interaction among GRF components measured by the four force sensors, this section proposes three set of features to characterize postural stability. Instead of studying the interdependency between ***F***_1_, ***F***_2_, ***F***_3_ and ***F***_4_, this work focuses on the coupling between the VGRF components, namely, *F*_1*z*_, *F*_2*z*_, *F*_3*z*_ and *F*_4*z*_. This decision is based on the following reasons. First, three-dimensional force sensors are much more expensive than one-dimensional force sensors. Therefore, developing postural stability features that can be derived directly from the VGRF components can significantly reduce the cost of the measurement system. Second, correlation measures between scalar variables are much simpler to develop than correlation measures between vectors. Third, during quiet standing, the VGRF is much larger than the GRF components in the AP and ML directions. As a result, the horizontal components of the GRF can be considered negligible in computing the COP for quiet standing [43, 44]. This is also the reason why that, even lacking the capability of measuring the horizontal components of the GRF, Nintendo Wii balance board has still been demonstrated to be a valid and reliable tool for assessing the performance of standing balance [45–47].

To quantitatively demonstrate the dominance of the VGRF in quiet standing, by denoting the energy of signal *F*_14*z*_–*F*_23*z*_ as *E*_*v*_ and the energy of *f*_*x*_ as *E*_*x*_, the ratio *E*_*v*_/(*E*_*v*_ + *E*_*x*_) can be used to quantify the contribution of the VGRF on the *COP*_*x*_. For the younger age group the sample mean of this ratio is about 0.991, whereas for the older age group the sample mean of this ratio is about 0.996. Similar results were obtained in the *y* direction. These data support the assumption that the position of the COP during quiet standing depends largely on the VGRF. Consequently, during the process of quiet standing, the coordinates of the COP can be approximated by

5 $$COP_{x} \approx d_{x} \frac{{(F_{14z} - F_{23z} )}}{{f_{z} }}$$

6 $$COP_{y} \approx d_{y} \frac{{(F_{12z} - F_{34z} )}}{{f_{z} }}$$

In studying finger force production, finger force correlation has been used to characterize the strength of inter-digit coupling [48, 49]. Similarly, to characterize the degree of coupling among the VGRF components, this work uses the Pearson product-moment correlation coefficient to quantify the strength of coupling. Considering the possibility that such coupling may be time-varying, this work divides the time response of these VGRF components into a number of time subintervals. For the *k*th time subinterval, the following correlation coefficient was computed for every pair of *F*_*iz*_ and *F*_*jz*_ for *i* = 1, …, 4 and *j* = *i* + 1, …, 4.

7 $$\rho_{ij} [k] = \frac{{V({\user2{F}}_{iz} [k],{\user2{F}}_{jz} [k])}}{{S({\user2{F}}_{iz} [k])S({\user2{F}}_{jz} [k])}}$$

where *V*(*X*, *Y*) represents the sample covariance between random variables *X* and *Y*, *S*(*X*) denotes the sample standard deviation of random variable *X*, and ***F***_*iz*_[*k*] signifies the sampled data vector associated with the *k*th time subinterval of *F*_*iz*_. With a sampling rate of 512 Hz and 0.125 s subinterval length, ***F***_*iz*_[*k*] is a 64-dimensional vector. Note that the selection of 0.125 s was determined by a trial-and-error process that tested many different subinterval lengths. The subinterval 0.125 s is chosen since it yields the best classification results for our experimental study.

To quantify the coupling strength between the VGRF components, the first feature set proposed in this work consists of the mean of the absolute value of *ρ*_*ij*_ and can therefore be expressed as

8 $$C_{ij} = \frac{1}{K}\sum\limits_{k = 1}^{K} {\left| {\rho_{ij} [k]} \right|}$$

where *K* is the number of time subintervals. In addition, the average value of these correlation strength features *C*_*ij*_ is used to represent the overall coupling among the four VGRF components. This overall correlation strength feature, *M*_*c*_, can be written as

9 $$M_{c} = \frac{1}{6}\sum\limits_{i = 1}^{4} {\sum\limits_{j = i + 1}^{4} {C_{ij} } }$$

To help understand the neurophysiological and biomechanical bases of the proposed correlation strength features, we consider the analogy between the postural balance problem and the finger force coordination problem. As noted in the “Background” section, force sharing among multiple fingers is a typical motor redundancy problem since many different combinations of finger forces can give the same total force. Discovering the coordination strategies that can eliminate or utilize the redundant degrees of freedom (DOFs) has thus become a central issue of motor control [50]. A possible approach for reducing such redundancy is through constraining the biomechanical DOFs. For example, rather than controlling each DOF independently, the DOFs can be neurally coupled. Consequently, the occurrence of significant correlation between finger forces has been hypothesized to reflect control of the fingers more as a single unit. Based on this concept, it has been suggested that finger force correlation should be high when a reduction in the controlled DOFs is permitted by the task conditions. In contrast, finger force correlation should be lower when the task demands larger finger decoupling. Hence, finger force correlation can mirror results for the dynamical DOFs in finger forces [51, 52]. Based on this very idea, the proposed correlation strength features can be used to characterize the degree of interdependence among the VGRF components.

As described in the previous section, in order to maintain postural balance, the human balance control system needs to adjust the COP in order to regulate the COM. The moving direction of the COP therefore changes with time. Consequently, the GRF correlation patterns may also be time varying. For example, from Eq. (5), a possible coordination strategy to move the COP along the positive *x*-direction is to increase *F*_1*z*_ and *F*_4*z*_ and decrease *F*_2*z*_ and *F*_3*z*_, simultaneously. This results in *ρ*_12_ < 0, *ρ*_13_ < 0, *ρ*_14_ > 0, *ρ*_23_ > 0, *ρ*_24_ < 0, *ρ*_34_ < 0. Similarly, from Eq. (6), the COP can be driven to move in the positive *y*-direction by increasing *F*_1*z*_ and *F*_2*z*_ and decreasing *F*_3*z*_ and *F*_4*z*_ at the same time. This leads to *ρ*_12_ > 0, *ρ*_13_ < 0, *ρ*_14_ < 0, *ρ*_23_ < 0, *ρ*_24_ < 0, *ρ*_34_ > 0. Note that in these two cases, the correlation coefficients *ρ*_12_, *ρ*_14_, *ρ*_23_ and *ρ*_34_ have different signs.

These correlation coefficients may vary with the movement of the COP. Therefore, the second feature set proposed in this work characterizes the changing speed of the correlation patterns. Specifically, by defining the zero-crossing points (ZCPs) as the time instant that the correlation coefficient *ρ*_*ij*_ changes sign, the changing speed feature for *ρ*_*ij*_ is defined as the number of ZCPs of *ρ*_*ij*_. By denoting the number of ZCPs of *ρ*_*ij*_ as *n*_*ij*_, the overall correlation pattern changing speed feature is defined as

10 $$N_{C} = \frac{1}{6}\sum\limits_{i = 1}^{4} {\sum\limits_{j = i + 1}^{4} {n_{ij} } }$$

In the remaining part of the manuscript, *n*_*ij*_ and *N*_*C*_ will be referred to as features of the correlation pattern changing speed.

As addressed in the “Background” section, with COM as the output and COP as the control variables, the postural balance system can be modelled as a feedback control system. One of the key performance measures of a feedback control system is its speed of response which depends on the system bandwidth, the saturation limits of the control variables as well as the maximum changing rate of the control variables, etc. Since different COP movements require different GRF coordination patterns, therefore a rapidly varying COP motion requires a fast GRF coordination pattern changing rate. Consequently, the proposed correlation pattern changing speed features have the potential to assess the response speed of the postural balance system.

During multi-finger tasks, previous studies have identified two types of finger interaction modes: enslaving and compensation [53, 54]. In the enslaving mode there is a positive correlation between the individual finger forces; in the compensation mode there is a negative correlation. The distinction between these two interaction modes can be illustrated by considering a simple force coordination problem where a person uses two fingers to push a cart. To stabilize the cart, the time responses of the two finger forces (*f*_1_ and *f*_2_) should be negatively correlated and thus united into a force-stabilizing synergy [55, 56]. In contrast, if the goal is to efficiently change the cart’s acceleration by preventing these two forces from canceling each other’s effect, the time responses of these of two forces should be positively correlated. To generalize this concept to the postural balance problem, by setting *G*_*ijz*_ = −*F*_*ijz*_, Eqs. (5) and (6) are rewritten as:

11 $$COP_{x} \approx d_{x} \frac{{(F_{14z} + G_{23z} )}}{{f_{z} }}$$

12 $$COP_{y} \approx d_{y} \frac{{(F_{12z} + G_{34z} )}}{{f_{z} }}$$

As shown by Eq. (11), by interacting in the compensation mode, negatively correlated *F*_14*z*_ and *G*_23*z*_ have the tendency to stabilize the *COP*_*x*_. In contrast, when interacting in the enslaving mode, positively correlated *F*_14*z*_ and *G*_23*z*_ tend to be more capable of producing rapid *COP*_*x*_ movement than negatively correlated *F*_14*z*_ and *G*_23*z*_.

To investigate the relative amount of time that the balance control system operates in these two interaction modes, the first member of the third proposed feature set is the ratio of time forces *F*_14*z*_ and *G*_23*z*_ interacted in the enslaving mode and is signified as *SMR*_*x*_. Similarly, the second member of the third feature set, *SMR*_*y*_, represents the ratio of time forces *F*_12*z*_ and *G*_34*z*_ is coordinated in the enslaving mode. Hereafter, these two features will be referred to as the enslaving mode ratio features.

For a multi-finger interaction task, enslaving reflects mechanical and neural connections among fingers, while compensation results from synergic control of fingers to stabilize their net output [55]. Therefore, the proposed features can shed some light on the strategy employed by the postural balance control system in coordinating the VGRF components. This topic will be explored in more detail in the “Discussion” section.

### Statistical and classification methods

Validity (accuracy) and reliability (repeatability) are two fundamental properties that need to assured for any measurement method. In this study, the test–retest reliability of the postural stability features was evaluated using the intra-class correlation coefficients (ICC) that compares within-subject variability with between-subject variability. In particular, among different versions of ICC, the ICC (2, 1) [57] was employed since it is the most frequently adopted ICC model for COP features [22].

To compare the accuracy of the tested features in detecting the aging effects, each of the tested features was independently used to classify the two age groups. In solving the binary classification problems, we chose the older age group as the positive class when the sample mean of the tested feature was larger in the older group than in the younger group. Otherwise, the younger group was selected as the positive class. In classifying any given sample data point, if its feature value was larger than a specified threshold, then the classifier assigned this data point to the positive class. Otherwise, this data point was classified to the negative class.

If a positive class sample data point was correctly classified as positive, it was counted as a true positive. Otherwise, it was counted as a false negative. Similarly, if a negative class sample data point was classified as negative, it was counted as a true negative. Otherwise, this classification result was counted as a false positive. The true and false positive rates of the overall classification results vary with the value of the threshold. By sweeping the threshold from the most positive to the most negative values, a receiver operating characteristic (ROC) curve can be generated by plotting the true positive rate as a function of the false positive rate. Because the area under the curve (AUC) of the ROC represents an estimate of the probability that the classifier ranks a randomly chosen positive example higher than a negative example, AUC has been considered to be a good performance index for classification methods [58]. In addition to the AUC, this study calculated the best accuracy of the ROC curve as the second efficacy measure for the tested features.

As noted previously, there were twelve measurements from twelve experimental trials (3 measurements/session × 4 sessions) for each subject. Since the variability of a sample mean can be reduced by increasing the sample size, the reliability of the tested features can be improved by replacing the original single measurement result with the average of several measurement results. In addition to improving reliability, this multiple-trial average method can improve the performance of the classifiers since the generalization capability of a classifier depends strongly on the training set size. As an example, by using the 3-trial averaging method, 220 sample data points can be generated for each subject (the number of possible combinations for selecting 3 objects from 12 objects is 220). Therefore, the 3-trial average method increases the training set size from 240 (12 measures/person × 20 persons) to 44,000 (220 measures/person × 20 persons) data points for both classes. Another advantage of the multiple-trial average method is that by comparing results obtained from different number of trials the influences of inter-trial variability on classification results can be investigated. Unless otherwise indicated, the experimental results presented in this manuscript were obtained using 3-trial average method. The influence of the number of trials used by the multiple-trial average method will be independently investigated in the final part of the experimental study.

## Results

Tables 1 and 2 summarize the mean and standard deviations of the correlation strength features *C*_*ij*_ and correlation pattern changing speed *n*_*ij*_, respectively. As shown in Table 1, the mean of the correlation strength features of the older group are all larger than those of the younger group. The results of Table 2 show that the mean of *n*_*ij*_ of the younger group are all larger than those of the older group. Next, the proposed features are compared to one of the most popular and effective COP postural stability features.

**Table 1:** Mean and standard deviations of the proposed correlation strength features

Features	Age group	
	Younger	Older
*C*_12_	0.782 ± 0.024	0.816 ± 0.025
*C*_13_	0.773 ± 0.024	0.825 ± 0.030
*C*_14_	0.801 ± 0.022	0.825 ± 0.026
*C*_23_	0.785 ± 0.018	0.813 ± 0.024
*C*_24_	0.793 ± 0.024	0.834 ± 0.032
*C*_34_	0.770 ± 0.021	0.805 ± 0.026

Values are mean ± standard deviation

**Table 2:** Mean and standard deviations of the proposed features of correlation pattern changing speed

Features	Age group	
	Younger	Older
*n*_12_	94.6 ± 10.9	73.8 ± 13.4
*n*_13_	86.3 ± 10.7	59.4 ± 14.2
*n*_14_	83.3 ± 12.1	67.6 ± 14.7
*n*_23_	91.7 ± 11.5	77.0 ± 14.1
*n*_24_	75.1 ± 11.3	55.5 ± 15.7
*n*_34_	101.1 ± 9.3	80.5 ± 14.9

Values are mean ± standard deviation. Unit for features *n*_*ij*_: times/30 s

The conventional COP features can be broadly divided into three groups, namely, displacement-related, velocity-related and frequency-related measures. This work only compares the proposed features to a COP velocity feature for the following reasons. First, the COP velocity is one of the most commonly used postural stability measures [22]. Second, numerous studies have demonstrated that the COP velocity is one of the most reliable and informative COP measures for postural steadiness [19, 21, 23, 59–62]. Third, COP velocity was found to be significantly correlated with the risk of falling among elderly [1, 4]. Traditionally, the COP velocity can be represented by the mean velocity (*MV*), mean velocity in the ML direction (*MV*_*ML*_) and mean velocity in the AP direction (*MV*_*AP*_). This study chose the *MV*_*AP*_ as the benchmark reference since the *MV*_*AP*_ is considered the most sensitive measure for postural control assessment related regulatory balance activity [13, 60, 63].

Considering the equally important roles of COP and COM in postural balance, this study also tests two recently proposed COM features which can clearly distinguish the difference between age groups [64]. By denoting *COM*_*acc*_ as the ratio of the GRF in the AP direction to the body mass of the participant, the first tested COM feature is the standard deviation of *COM*_*acc*_ divided by the body height. The second tested COM feature is the peak frequency domain power of the *COM*_*acc*_ divided by squared body height. Hereafter, these two COM features will be referred as *COM*_*acc*_-*SD* and *COM*_*acc*_-*PP*, respectively.

To compare to these three COP and COM features, this work chose the overall correlation feature *M*_*c*_, the overall correlation changing speed feature *N*_*c*_ and the enslaving mode ratio feature *SMR*_*x*_ as the representative features for the first, second and third feature sets, respectively. In the remaining part of this manuscript, *M*_*c*_, *N*_*c*_ and *SMR*_*x*_ will be referred to as the core correlation features.

Table 3 summarizes the mean and standard deviations of *MV*_*AP*_, two tested *COM*_*acc*_ features, the core correlation features and the enslaving mode ratio *SMR*_*y*_. In agreement with previous studies [13], the experimental results here indicate that aging increases the COP velocity. As shown in Table 3, the older age group has larger *MV*_*AP*_, *COM*_*acc*_-*SP*, *COM*_*acc*_-*PP*, *M*_*c*_, *SMR*_*x*_ and *SMR*_*y*_ but smaller *N*_*C*_ than the younger group. The results of Table 4 demonstrate that all three core correlation features have higher accuracy and larger AUC than the three tested COP and COM features.

**Table 3:** Mean and standard deviations of the COP feature *MV*_*AP*_, core correlation features and enslaving mode ratio feature *SMR*_*y*_

Features	Age group	
	Younger	Older
*MV*_*AP*_	5.45 ± 1.06	7.28 ± 2.00
*COM*_*acc*_-*SD*	3.36 ± 0.87 × 10^−2^	4.28 ± 1.31 × 10^−2^
*COM*_*acc*_-*PP*	3.32 ± 0.56 × 10^−4^	3.82 ± 0.69 × 10^−4^
*M*_*c*_	0.784 ± 0.019	0.819 ± 0.025
*N*_*c*_	88.7 ± 8.8	69.0 ± 13.0
*SMR*_*x*_	0.750 ± 0.065	0.881 ± 0.044
*SMR*_*y*_	0.522 ± 0.081	0.596 ± 0.062

Values are mean ± standard deviation

Units of features are as follows: mm/s (*MV*_*AP*_), times/30 s (*N*_*c*_)

**Table 4:** Accuracy and AUC for the COP feature *MV*_*AP*_ and the core correlation features

Features	AUC	Accuracy
*MV*_*AP*_	0.778	0.733
*COM*_*acc*_-*SD*	0.723	0.672
*COM*_*acc*_-*PP*	0.719	0.703
*M*_*c*_	0.865	0.779
*N*_*c*_	0.897	0.801
*SMR*_*x*_	0.952	0.864

**Fig. 2:**
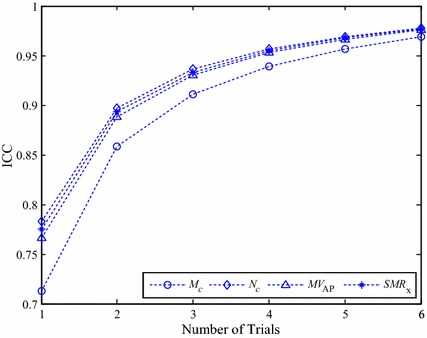
Intra-class correlation coefficients of COP feature *MV*_*AP*_ and the core correlation features obtained by the multiple-trial average method

**Fig. 3:**
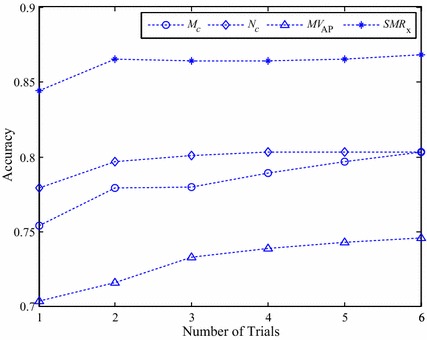
Classification accuracy of COP feature *MV*_*AP*_ and the core correlation features obtained by the multiple-trial average method

To investigate how many trials must be averaged to obtain reliable and accurate measurement results, the ICC values and the results of classification accuracy obtained by the multiple-trial average method were plotted in Figs. 2 and 3, respectively. These two figures show a general trend that the reliability and classification accuracy improve as the number of trials increases. As shown in Fig. 2, the resulting ICC curves of *MV*_*AP*_, *N*_*c*_ and *SMR*_*x*_ are quite similar. In comparison, the reliability of *M*_*c*_ is not as good as the other three features. The results of Fig. 3 show that, regardless of the number of trials, all three core correlation features were able to achieve better classification accuracy than *MV*_*AP*_.

## Discussion

The results of Tables 1, 2 and 3 show that aging intensifies the coupling strength among the VGRF components, reduces the changing speed of the correlation patterns and increases the ratio of time of enslaving mode. To analyze the results associated with the correlation strength features, we have considered the analogy between the postural balance problem and the finger force coordination problem. As noted in the “Background” section, under the constraints of the task, higher finger force correlation is associated with the reduction in the controlled DOFs of fingers and lower finger force correlation is related to larger finger decoupling. Hence, finger force correlation can mirror results for the dynamical DOFs in finger forces [51, 52].

For the postural balance problem, the first two task constraints are the COP coordinate Eqs. (5) and (6). The third constraint comes from the force balance in the vertical direction,

13 $$F_{1z} + F_{2z} + F_{3z} + F_{4z} = M(g + a_{z} )$$

where *M* is the mass of the human body, *g* is gravity and *a*_*z*_ is the vertical acceleration of the mass center of the human body. With Eqs. (5), (6) and (13) as the task constraints, the coordination of the four VGRF components can therefore be regarded as a redundant DOF problem whose goal is to identify how a person copes with redundancy during quiet standing. As shown by the comparative results of the correlation strength features summarized in Table 1, aging increases the level of coupling among VGRF components. The results imply that aging leads to restricted dynamical DOFs and thus constrains the older age group’s capability in manipulating COP which in turn results in poor postural stability.

The results of Table 2 indicate that the features of correlation pattern changing speed of the younger group is larger than those of the older group. This may be associated with the aging-related decline of the speed of response of the balance control system. Such a decline may be attributed to the deterioration of the speed and organization of muscle force [65], decline in nervous system speed [66] and progressive decrease of sensory input [67]. Since all these age-induced factors can degrade the ability to respond quickly to a loss of balance and increase the risk of falling, the possible associations between these aging-related impairments and the proposed features of correlation pattern changing speed warrant further study.

Regarding the behavior of the enslaving mode ratio features *SMR*_*x*_ and *SMR*_*y*_, the results of Table 3 show that both age groups spent more time in the enslaving mode than in the compensation mode. The results of Table 3 also show that the older age group spent more time in the enslaving mode than the younger group. Such results can be interpreted by considering the connection between the COP and VGRF components. As addressed in the “Methods” section , any difference between the vertical projection of the COM and the COP will result in a moment that can destabilize the balance of the body. Therefore, the balance control system needs to regulate the COM by moving the COP. To keep the COM within a desired region between the two feet, the COP oscillates on either side of the moving COM at higher frequencies. Since the enslaving mode represents a more efficient choice for moving the COP than the compensation mode, it is reasonable that most of the time (about 75 % for the younger group and 88 % for the older group) forces *F*_14*z*_ and *G*_23*z*_ interacted in the enslaving mode. However, if the AP component of the COM coincides with *COP*_*x*_ or the difference is too small to be detected by the human sensory system, then *F*_14*z*_ and *G*_23*z*_ interact in the compensation mode since negative co-variation of these two forces produces a stabilizing effect on *COP*_*x*_. This stabilizing effect leads directly to small *MV*_*AP*_. A similar argument applies to ML direction. Since it is well known that a smaller COP velocity represents better balance stability, this explains why the younger age group spent more time in the compensation mode than the older group.

The results of Table 3 also show that *SMR*_*y*_ is considerably less than *SMR*_*x*_ (about 52 % for the younger group and 60 % for the older group). This indicates that, compared to the forces *F*_14*z*_ and *G*_23*z*_ of the AP direction, forces *F*_12*z*_ and *G*_34*z*_ of the ML direction spent more time in the compensation mode. Such a difference between *SMR*_*x*_ and *SMR*_*y*_ can be explained based on several previous findings. First, it is well known that the posture balance control strategies for the AP and ML directions are fundamentally different. By simultaneously loading one limb and unloading the opposite limb to shift the COP, the control policy of the ML direction has been identified as a hip “load/unload” strategy while the AP control was independent and controlled at the ankle [17]. Second, as the feet were positioned well apart, the stance in the ML direction was considered to be particularly stable [68, 69]. This observation is supported by the experimental results that the *COP*_*x*_ typically has larger range, path length and velocity than those of the *COP*_*y*_ [13, 70, 71]. It was also found that COP features in the AP direction were generally more variable than those in the ML direction [72]. The results on *SMR*_*x*_ and *SMR*_*y*_ are considered in agreement with these findings since by spending more time in the compensation mode, forces *F*_12*z*_ and *G*_34*z*_ provide a more stabilizing effect on the *COP*_*y*_ than forces *F*_14*z*_ and *G*_23*z*_ can provide for the *COP*_*x*_.

Since human balance performance is not perfectly repeatable and thus may vary from trial to trial, the variability can degrade the performance of the postural stability features in assessing balance ability. Therefore, an important property that also needs to be examined is the test–retest reliability of the postural stability features. In this work, we used ICC (2, 1) as the reliability metric to quantify the degree of agreement between measurement results across trials. Theoretically, the value of ICC varies from 0 (no reliability) to 1 (perfect reliability). To determine the number of trials required by the multiple-trial average method to achieve a satisfactory ICC, one can incrementally increase the number of trials until the value of ICC plateaus. Since adding extra trials increases the time and effort required for the experimental tests, an efficient feature should be able to satisfy the reliability requirement by using the averages of a small number of trials.

The value of ICC was interpreted using the following criteria: 0.00–0.39 poor, 0.40–0.59 fair, 0.60–0.74 good and 0.75–1.00 excellent [73]. With these criteria, the results of Fig. 2 show that the COP feature *MV*_*AP*_ and the proposed features *N*_*c*_ and *SMR*_*x*_ can achieve excellent reliability with single-trial measurements. The proposed features *N*_*c*_ and *SMR*_*x*_ provide a level of reliability comparable to that of *MV*_*AP*_, which was considered as one of the most reliable COP features. In addition, for the four features shown in Fig. 2, their ICC values are all larger than 0.9 when the number of trials increases to 3 or higher.

Despite the fact that *MV*_*AP*_, *N*_*c*_ and *SMR*_*x*_ provide comparable ICC test results, comparing the classification performances of these features is still considered to be indispensable since reliability is only a necessary but not a sufficient condition for validity. The results of Fig. 3 show that, compared to *MV*_*AP*_, all three core correlation features can achieve higher classification accuracy with a smaller number of trials. In particular, as shown by Table 4, with single-trial measurements, the classification accuracy obtained by *MV*_*AP*_, *M*_*c*_, *N*_*c*_ and *SMR*_*x*_ are 0.733, 0.779, 0.801 and 0.864, respectively. In comparison, by increasing the number of trials to six, the multiple-trial average method improved the classification accuracy of *MV*_*AP*_ to 0.746. This is still inferior to the classification accuracy obtained by the core correlation features that used only single trial averages.

Figure 3 shows that in applying the multiple-average method the proposed features *N*_*c*_ and *SMR*_*x*_ plateau when the number of trials increases to two. In contrast, to approach the plateau level, the number of trials required by *M*_*c*_ and *MV*_*AP*_ must be four or larger. Regarding reliability, with 2-trial averages, the values of ICC(2, 1) for these two features are close to 0.9. These results indicate that in terms of the capability in distinguishing the aging effects on postural balance *N*_*c*_ and *SMR*_*x*_ are more robust to inter-trial variability than *M*_*c*_ and *MV*_*AP*_. This advantage of robustness is a very attractive property since one can reduce the time and cost of the measurement process by requiring fewer experimental trials.

One possible direction of future work is to perform in-depth studies of the proposed features. These include, but are not limited to, investigating the associations between the proposed features and conventional postural stability features, studying the performance of the proposed features in characterizing the postural stability for different stance conditions and examining the connections between the proposed features and standard clinical balance measures such as BBS. Another direction for possible future work is to explore the potential applications of the proposed features. These may include predicting the risk of falling for the aged population, characterizing the severity of balance disorders, progressive monitoring of the outcomes of rehabilitation designed to improve the functional capabilities of balance control and studying the impacts of other pathologies on balance ability.

## Conclusion

By extracting information from the correlation patterns of the VGRF components, this study proposes three sets of features to assess human postural stability during quiet standing. The first set of features quantifies the coupling strength among the VGRF components. The second set of features characterizes the changing speed of the correlation patterns. By comparing the amount of time the VGRF components interacted in enslaving (positive correlation pattern) and compensation modes (negative correlation pattern), the third set of features evaluates the COP stabilizing effect produced by the VGRF components. In addition to experimentally demonstrating the reliability of the proposed features, the efficacy of the proposed features has also been tested by using them to classify two age groups (18–24 and 65–73 years) in quiet standing. Experimental results show that aging intensifies the coupling strength among the VGRF components, reduces the changing speed of the correlation patterns and increases the ratio of time of enslaving mode. In addition, the proposed features are not only robust to inter-trial variability but also more accurate than one of the most effective COP features and two recently proposed COM features in classifying the older and younger age groups. An additional advantage of the proposed approach is that it reduces the force sensing requirement from 3D to 1D, substantially reducing the cost of the force plate measurement system.

## Abbreviations

AP: anterior–posterior
AUC: area under the curve
BBS: Berg balance scale
COM: center of mass
COP: center of pressure
DOF: degree of freedom
GRF: ground reaction force
ICC: intra-class correlation coefficients
ML: medial–lateral
MV: mean velocity
MV_AP_: mean velocity in the AP direction
MV_ML_: mean velocity in the ML direction
ROC: receiver operating characteristic
VGRF: vertical ground reaction force
ZCP: zero-crossing point
